# Integrating a focus on health equity in implementation science: Case examples from the national cancer institute’s implementation science in cancer control centers (ISC^3^) network

**DOI:** 10.1017/cts.2023.638

**Published:** 2023-09-29

**Authors:** Kelly A. Aschbrenner, April Y. Oh, Rachel G. Tabak, Peggy A. Hannon, Heather E. Angier, W. Todd Moore, Sonja Likumahuwa-Ackman, Jennifer K. Carroll, Ana A. Baumann, Rinad S. Beidas, Stephanie Mazzucca-Ragan, Erika A. Waters, Rajani S. Sadasivam, Rachel C. Shelton

**Affiliations:** 1 Department of Psychiatry, Geisel School of Medicine at Dartmouth, Dartmouth-Hitchcock Medical Center, Hanover, NH, USA; 2 Division of Cancer Control and Population Sciences, National Cancer Institute, Rockville, MD, USA; 3 Brown School, Washington University in St. Louis, St. Louis, MO, USA; 4 Department of Health Systems and Population Health, School of Public Health, University of Washington, Seattle, WA, USA; 5 Department of Family Medicine, Oregon Health & Science University, Portland, OR, USA; 6 Department of Family Medicine, University of Colorado, Aurora, CO, USA; 7 Division of Public Health Sciences, Department of Surgery, Washington University School of Medicine, St. Louis, MO, USA; 8 Department of Medical Social Sciences, Northwestern University Feinberg School of Medicine, Chicago, IL, USA; 9 Division of Health Informatics and Implementation Science, Department of Population and Quantitative Health Sciences, University of Massachusetts Chan Medical School, Worcester, MA, USA; 10 Department of Sociomedical Sciences, Mailman School of Public Health, Columbia University, New York, NY, USA

**Keywords:** Cancer control, implementation science, health equity, capacity building, community engagement

## Abstract

**Background:**

A Health Equity Task Force (HETF) of members from seven Centers funded by the National Cancer Institute’s (NCI) Implementation Science in Cancer Control Centers (ISC^3^) network sought to identify case examples of how Centers were applying a focus on health equity in implementation science to inform future research and capacity-building efforts.

**Methods:**

HETF members at each ISC^3^ collected information on how health equity was conceptualized, operationalized, and addressed in initial research and capacity-building efforts across the seven ISC^3^ Centers funded in 2019–2020. Each Center completed a questionnaire assessing five health equity domains central to implementation science (e.g., community engagement; implementation science theories, models, and frameworks (TMFs); and engaging underrepresented scholars). Data generated illustrative examples from these five domains.

**Results:**

Centers reported a range of approaches focusing on health equity in implementation research and capacity-building efforts, including (1) engaging diverse community partners/settings in making decisions about research priorities and projects; (2) applying health equity within a single TMF applied across projects or various TMFs used in specific projects; (3) evaluating health equity in operationalizing and measuring health and implementation outcomes; (4) building capacity for health equity-focused implementation science among trainees, early career scholars, and partnering organizations; and (5) leveraging varying levels of institutional resources and efforts to engage, include, and support underrepresented scholars.

**Conclusions:**

Examples of approaches to integrating health equity across the ISC^3^ network can inform other investigators and centers’ efforts to build capacity and infrastructure to support growth and expansion of health equity-focused implementation science.

## Background

Over the past twenty years, the field of implementation science has contributed to identifying and understanding barriers and facilitators to implementing evidence-based practices (EBPs) in healthcare and public health service and practice and has generated evidence for effective strategies to improve adoption, implementation, sustainment, and scale-up of EBPs [1,2]. Despite substantial progress in the science of implementing EBPs, widespread inequities in healthcare delivery and health outcomes linked with underlying social, structural, economic, and racial injustices persist [3]. Implementation research that concentrates on explicitly understanding and addressing factors driving inequities and disparities holds promise for advancing health equity [4].

Health equity is the absence of avoidable, unfair, or remediable differences in health [5,6]. It is the principle underlying a commitment to reduce and ultimately eliminate a health disparity and its determinants [7]. There are numerous historical and ongoing systemic and structural drivers and systems that disproportionately create and maintain social and health inequities, particularly for people from marginalized racial and ethnic groups; people with disabilities; immigrant and refugee communities; people who are LGBTQI+ (lesbian, gay, bisexual, transgender, queer, and intersex); and people with limited English proficiency in the U.S. context [8]. Additionally, contextual factors such as where people live and the physical and social environment contribute to and exacerbate health inequalities [9,10]. Even well-intentioned, large-scale efforts to implement, sustain, and scale up EBPs, and other innovations may disproportionately benefit privileged groups and settings, particularly when reach and uptake are limited among settings and populations that experience numerous structural disadvantages to health and healthcare [11].

Implementation scientists are increasingly prioritizing an explicit focus on health equity in implementation research [4,12,13]. This focus is aligned with the recognition that addressing inequities in implementation research and practice at multiple contextual levels (e.g., individual, clinical, community, policy) is critical to being able to effectively address the research-to-practice gap [14]. While interest continues to grow in applying equity approaches in implementation science, many investigators in the field are inexperienced with regard to how to operationalize or sustain a focus on health equity in their research [15]. Key questions and recommendations for grounding implementation research in health equity are provided in the literature [16], with some guidance for integrating a focus on equity in implementation frameworks and methods [4,16–19]. However, with some exceptions [20,21], there are limited instructive examples that can guide or inform the field. This manuscript seeks to address this gap by providing case examples of integrating and applying a focus on health equity across a national network of implementation science research Centers to inform other investigators’ and Centers’ efforts to build capacity and infrastructure to advance health equity in implementation science.

## Materials and Methods

### Overview of the Implementation Science Network

The National Cancer Institute (NCI) funded seven Implementation Science in Cancer Control Centers (ISC^3^) in fiscal years 2019-2020 (Table 1). Funding was part of the NCI Cancer Moonshot^SM^ [22], an investment in funding by the US government focusing on areas of cancer research with high potential for patient impact. The ISC^3^ network consists of research Centers that support and advance the development, testing, and refinement of innovative approaches to implement a range of EBPs across the cancer control continuum [23]. ISC^3^ seeks to enhance the capacity of researchers, practitioners, and communities to apply implementation science approaches, methods, and measures. All Centers feature implementation laboratories (I-Labs) to engage clinical and community partners in research and capacity-building efforts [24]. Advancing health equity in cancer prevention and control among historically underserved populations through implementation science was a potential theme area for Centers highlighted in the Funding Opportunity Announcement (RFA-CA-19-006) and the organization framework for the Centers [23].

**Table 1.** The ISC^3^ program is composed of seven Centers funded by RFA-CA-19-005 and RFA-CA-19-006

Center	PI/MPI*	Institution
The Implementation Science Center for Cancer Control Equity (ISCCCE)	Karen M. Emmons, Ph.D. Elsie M. Taveras, M.D., M.P.H.	Harvard T.H. Chan School of Public Health
Building Research in Implementation and Dissemination to close Gaps and achieve Equity in Cancer Control Center (BRIDGE-C2)	Jennifer E. DeVoe, M.D., D.Phil. Heather E. Angier, Ph.D. Nathalie Huguet, Ph.D.	Oregon Health & Science University
Colorado Implementation Science Center in Cancer Control (COISC3)	Russell E. Glasgow, Ph.D.	University of Colorado School of Medicine
Optimizing Implementation in Cancer Control (OPTICC)	Bryan J. Weiner, Ph.D. Peggy Hannon, Ph.D., M.P.H. Cara C. Lewis, Ph.D.	University of Washington
Implementation and Informatics – Developing Adaptable Processes and Technologies for Cancer Control (iDAPT)	Kristie Long Foley, Ph.D. Thomas K. Houston II, M.D., M.P.H. Sarah L. Cutrona, M.D., M.P.H.	Wake Forest University School of Medicine/University of Massachusetts Chan Medical School
Washington University Implementation Science Center for Cancer Control (WU-ISCCC)	Ross C. Brownson, Ph.D. Graham A. Colditz, M.D., Dr.PH.	Washington University in St Louis
Penn Implementation Science Center in Cancer Control (Penn ISC3)	Rinad S. Beidas, Ph.D. Justin Bekelman, M.D. Robert Schnoll, Ph.D.	University of Pennsylvania/Northwestern University

* These were the Center PI/MPIs at the time the survey was conducted in January of 2022.

### Survey Development and Administration

Health Equity Task Force (HETF) members representing each of the seven ISC^3^ Centers used a consensus-based process [25,26] to identify five overarching domains (Table 2) identified in the implementation science literature as central to focusing on health equity in implementation science [4,12,16,27]. These domains informed the development of a questionnaire to assess how health equity was addressed, conceptualized, and operationalized across the Centers during the initial launching of the ISC^3^ network. To minimize burden, the questionnaire instructed respondents to report information pertaining to key domains most relevant to their respective Center, rather than responding to every item. The HETF team piloted and refined the questionnaire based on feedback from one Center (Penn ISC3). HETF members then worked with investigators and Center PIs from their respective centers to complete the questionnaire. Data were collected across centers between January 2022 and March 2022. The Dartmouth Health Human Research Protection Program made a formal determination of “not human subjects research” for this research.

**Table 2.** Health equity domains investigated in the ISC^3^ survey

Domain	Definition	Survey question
1) Community engagement in health equity-focused implementation research	Promote multi-level, diverse stakeholder or community engagement in health equity-focused implementation research	*Describe how stakeholders are engaged and their activity, role, responsibility, and impact on projects.* 1. How did your project define and identify your stakeholders? Discuss your stakeholder’s role in defining health equity. 2. How, when, and to what extent are stakeholders or community partners engaged in the project? 3. What have you learned from your community/stakeholders? Both specific to health equity and more broadly? 4. What (if any) additional supports would be beneficial to facilitate bidirectional engagement with stakeholders in working towards promoting health equity? 5. What challenges or lessons learned do you have to share in this area?
2) Emphasizing health equity in the application of implementation science theories, models, and frameworks	Emphasize or integrate health equity when identifying and/or using theories, measures, and frameworks	*How are health equity-related systems, social, and structural determinants operationalized and measured in your research projects?* 1. Where are you in this process of integrating equity into TMF (e.g., in development, launched, empirically testing in process, completed, etc.)? 2. Which TMF(s) (from implementation science or outside our field) is your center using related to health equity? Which TMFs are you finding the most useful/challenging? 3. How are TMFs being applied to assess contextual determinants? How are they informing interventions or implementation strategies, including refinements or adaptations? 4. What challenges or lessons learned do you have to share in this area?
3) Evaluating health equity in implementation processes and outcomes	Amplify focus on health equity in the evaluation of implementation outcomes	*How do you measure or track health equity?* 1. What tools or models is your center utilizing to guide evaluation or measurement (e.g., Proctor model, RE-AIM, etc.)? 2. What social dimensions are you using to track health equity metrics (e.g., disability, race and ethnicity, socioeconomic, setting/clinic, gender, geography)? 3. Discuss any unintended outcomes of your projects on stakeholders or historically underrepresented faculty. Has your project had unintended consequences in exacerbating inequities or health equity-related outcomes? 4. What challenges or lessons learned do you have to share in this area?
4) Building capacity for focusing on health equity in implementation science	Build capacity for applying health equity in implementation research across career stages	*Describe how you build capacity for researchers to apply health equity within your ISC* ^*3*^ *Center.* 1. What training, webinars, and other resources on implementation science with a health equity focus are offered by your center? 2. What would priority areas for capacity building be for researchers? For community partners/labs? 3. Are there examples of mentorship provided through your Center including a health equity focus in implementation science/cancer-related grants? 4. What challenges or lessons learned do you have to share in this area?
5) Engaging and including underrepresented scholars in implementation science	Identify opportunities for engaging underrepresented scholars in implementation science.	*Please describe the best practices, promising approaches, and/or policies that your center uses to engage, recruit, retain, promote, and support underrepresented scholars.* 1. When and where does your Center’s “career training pathways” begin? 2. How do you identify equitable hiring practices (recruitment, retention support) and ensure their consistent use? 3. How do you evaluate projects/activities that support recruitment, promotion, and retention of diverse scholars? 4. What have you learned from diverse scholars on recruitment, promotion, and retention practices at your center? 5. Do you have successes or examples of mentoring historically underrepresented scholars (e.g., funded grants, paper, collaborations, etc.)? 6. What challenges or lessons learned do you have to share in this area?

Once data was collected, five HETF members read the completed questionnaires from each of the seven Centers to identify illustrative examples, challenges, and lessons learned within and across each of the five domains. Two members (KAA and RCS) then synthesized the information and organized the key findings, using member checking with Principal Investigators and HETF members from each of the seven Centers to ensure accuracy of the findings.

## Results

In the following sections, we summarize key survey findings and provide two illustrative examples from each of the five survey domains. Further details on findings, including data not shown in the text, are provided in Supplementary File 1.

### Domain 1: Community Engagement in Health Equity-Focused Implementation Research

Centers reported engaging and collaborating with a wide variety of healthcare networks, systems, providers, and patients to focus explicitly on health equity within implementation research. This often involved active engagement with healthcare networks perceived as having strong potential to advance equity efforts, including primary care associations, rural research networks, and federally qualified health centers (FQHCs). To help ensure issues related to health equity were understood and centered in the research, many Centers sought to actively engage and elevate the voices and perspectives of clinical and community partners throughout various phases of research, including through reviewing proposed research and/or planning or conducting and disseminating research.

Example 1.1 BRIDGE-C2: “*The I-Lab at the BRIDGE-C2 Center formalized collaboration with OCHIN’s Health Resources and Services Administration-funded Health-Centered Controlled Network (HCCN). [OCHIN is a non-profit healthcare innovation center]…The HCCN Network is focused on quality improvement (QI) for federally qualified health centers (FQHCs) and look-alikes to aid in health equity. These connections provide the BRIDGE-C2 Center with strategic alignment around knowledge of QI initiatives (e.g., interactive colorectal cancer screening decision tree) and electronic health record enhancements (e.g., colorectal cancer and cervical cancer screening Health Maintenance Topics). These connections also allow the Center to leverage resources across teams for evaluation of specific interventions, survey activities and to access a cohort of clinics for deeper investigation (e.g., qualitative interviewing), and understand community needs around health equity*. *For example, in May 2020, HCCN and BRIDGE-C2 co-designed an evaluation tool and conducted interviews with OCHIN staff about their technical assistance and QI efforts and the impacts that COVID-19 had on their direct work with community health centers. The I-Lab connects with HCCN and clinical team members through monthly meetings. This bidirectional support and community building at the leadership level offers multi-level engagement with downstream impacts particularly around health equity.”*

Centers actively sought partner input using a wide range of methods and approaches to facilitate engagement, including pre-implementation needs assessments, key informant interviews, focus groups, user-centered design processes, feedback, and input on proposed pilot studies, as well as informal conversations. Some centers also reported that partner input and voice shaped the content and design of interventions and implementation strategies to advance health equity by integrating or adapting to their interests, priorities, and preferences on the intervention components. This included key aspects such as the length, language, terminology, and platforms used to deliver interventions and implement more equitably.

Example 1.2 Harvard ISCCCE: “*In the first round of ISCCCE’s cancer prevention and control pilots, the I-Lab played a significant role in engaging Community Health Center (CHC) stakeholders by providing support and technical assistance as they implemented a paired cancer screening and social determinants of health pilot. This work has included building capacity and engagement with community members and/or community-based organizations (CBOs). As an example*, *I-Lab staff worked directly with CHC stakeholders throughout the implementation process, starting with a needs assessment with CHC teams to learn about their response to COVID, how they were addressing social determinants of health and learning about their current FIT [colorectal cancer screening] test workflows. In the guided adaptation phase of the methods pilot, the I-Lab provided implementation facilitation to CHCs to conduct data comparisons of outreach and use of the intervention. This included individuals reached for colorectal cancer screening and those who completed screening through the pilot implementation phase with the goal of identifying population groups not being reached or screened with current workflows (for example, by language or race) in order to modify strategies to reach those groups*.”

### Domain 2: Emphasizing Health Equity in Application of Implementation Science Theories, Models, and Frameworks (TMFs)

We asked Centers to identify how they were applying and operationalizing theories, models, or frameworks (TMFs) addressing health equity in the research projects at their Centers, including modifying existing implementation science TMFs to bring a health equity focus, or identifying TMFs from outside the field to bring an explicit health equity focus. We found that Centers were taking variable approaches to applying TMFs with a focus on equity, with some integrating a single TMF across the projects and others applying to specific aspects of pilots. A wide range of TMFs were being applied to center equity in the context of research projects, including the Health Equity Implementation Framework [19] and an adapted version of the Consolidated Framework for Implementation Framework [28]. Several of the Centers were applying TMFs to enhance understanding of equity-focused determinants to inform implementation strategies; examples of other frameworks applied include the Practical, Robust Implementation and Sustainability Model (PRISM) [29] and the Practice Change Model [30].

Centers reported applying TMFs to guide assessment of implementation outcomes with a focus on equity including tracking equitable/inequitable adoption, reach, and implementation with the Reach, Effectiveness, Adoption, Implementation, Maintenance Framework (RE-AIM)[31]. Finally, in response to a noted gap in implementation science TMFs that integrate health equity, a cross-ISC^3^ activity expanded the widely used web tool https://dissemination-implementation.org/ to help research and evaluation teams select, adapt, combine, use, and assess TMFs. The expansion led by the Colorado and Washington University ISC^3^s includes a new section on health equity that provides concrete examples of applying different TMFs to address equity. The webtool also integrates equity centrally into the resource by 1) coding the existing TMFs on a health construct and 2) adding key health equity TMFs to the webtool and coding them on dissemination and implementation constructs.

Example 2.1 WU-ISCCC: *“In a recently-funded project, a researcher from the Center will use the Health Equity Implementation Framework [19] to guide work to quantify and characterize the direct costs, including the financial burden from insurance-related fees (e.g., co-pays, co-insurance) and indirect costs, including the unforeseen costs (e.g., loss of work, absenteeism, presenteeism), to support the implementation of shared decision-making among Black men with prostate cancer as they weigh the tradeoffs between treatment options.”*

Example 2.2 Penn ISC3: “*Our approach towards operationalizing health equity-related measures into the Penn ISC3 corpus of work is broadly informed by several implementation science frameworks. The Consolidated Framework for Implementation Research (CFIR) [28] determinants framework broadly informs our focus on both the Outer and Inner Contextual levels that shape health inequities and inequitable implementation. Additionally, we will expand upon potential equity-related determinants that shape these inequities by examining contextual, provider, and patient-level factors that might explain these findings (e.g., why were nudges inequitably adopted/effective across different racial/ethnic or socioeconomic groups among patients? Why might providers differentially refer patients?), informed by The Health Equity Implementation Framework [19]. Specifically, we will oversample patients by race and income to explore these differences. Additionally, in the signature pilot projects, we are integrating CFIR with the RE-AIM Extension for Equity and Sustainability [31] to inform selection of our implementation indicators and outcomes and tracking equitable/inequitable adoption, reach, and implementation in relation to behavioral nudges, as well as potential differences in patient perceptions of acceptability and appropriateness of implementation strategies (by income and race/ethnicity). Challenges here have been having sufficient variation in patient/setting characteristics to examine some of these differences; additionally, it can be challenging to adequately capture some of the contextual factors that may be at play (e.g., provider or system biases*).”

### Domain 3: Evaluating Health Equity in Implementation Processes and Outcomes

Centers reported operationalizing a wide range of health and implementation outcomes and evaluating research projects with an explicit focus on health equity. This focus often related to identifying when/where there were gaps in implementation occurring across the settings and populations in which centers were engaged in research (e.g., in processes, referrals, screening, access, strategy delivery). Additionally, several Centers reported tracking gaps in both reach and representation at individual and setting level along the implementation continuum and across a range of social and setting dimensions (e.g., language, race, ethnicity, income, geography).

Example 3.1 iDAPT “*One of our investigators is developing a ‘Usable Measure for Digital Divide in the Clinical Setting’ as a methods pilot study. Digital tools can improve effectiveness and reduce healthcare barriers. However, to fully realize their potential (to broaden reach to all populations), implementation strategies must address the digital divide challenge. The digital divide for health-related technology may be a result of various factors. At the person level, it can include motivation, personality traits (e.g., openness, extraversion, conscientiousness), and digital skills. Social determinants of health also impact the digital divide. At the population level, this understanding will allow implementers to develop the appropriate strategies to ameliorate or eliminate disparities. Understanding these factors and their interactions is helpful for clinical implementers to address the digital divide. Our first goal is to identify electronic health record ‘markers’ for measures for the digital divide. These markers can be augmented with questions (as deemed appropriate) to allow clinical implementations to better measure and address the value of digital tools during clinical implementation*.”

Example 3.2 Colorado ISC3: ***“*** *The aim of the pilot is to conduct a feasibility trial of the implementation of a patient-centered shared decision-making and lung cancer screening (LCS) strategy in rural primary care clinics in Colorado. This intervention package includes a formal shared decision-making process and smoking cessation support for rural primary care clinics that is aligned with Center for Medicare and Medicaid Services criteria for LCS. We are using the PRISM [32] and RE-AIM frameworks [31,33] to guide our evaluation – as well as planning and implementation. Importantly we are focusing on issues of both: a) representation (who is involved in planning, execution, and evaluation at each of the RE-AIM “steps”); and b) representativeness (or equity) of outcomes on each RE-AIM dimension to evaluate the cumulative or “Cascade” effect of potential inequities at each point. Trying to comprehensively assess all potential equity-related factors, and especially context is an overwhelming task. For example, on each RE-AIM dimension we are assessing representativeness but on what specific factors and how many factors are a challenge. These decisions are based on both the literature and on factors from our experience that are most likely to be strongly related to outcomes, as it is impossible to address everything.”*

### Domain 4: Building Capacity for Focusing on Health Equity in Implementation Science

Building capacity for applying and prioritizing a focus on health equity in implementation science was discussed in relation to (1) research training and mentoring for trainees, early career and established investigators, and members of partnering organizations; and (2) developing and disseminating tools and resources to advance application of health equity in implementation science. Specific strategies to build capacity ranged from research tools to changing organizational policies. These included (1) providing access to funding and consultation for pilot grants focusing on health equity in implementation science; (2) positioning community partners as co-investigators on pilot grants related to health equity; (3) creating tools that can be widely used to support a focus on health equity in implementation science (e.g., bibliography of existing readings on health equity from experts outside implementation science); (4) developing organizational policies that reinforce and encourage invitation of community partners to serve as manuscript co-authors; and (5) increasing research center and institutional knowledge, attitudes and awareness by conducting seminars and meetings to develop and disseminate health equity-focused implementation research and products. Many capacity-building initiatives and strategies leveraged existing resources and training programs at the university or across the implementation science community (e.g., establishing a partnership with existing training programs that focus on enhancing diversity and health equity at the university through mentorship and research training).

Example 4.1 WU-ISCCC: “*In our Center, the main ways that we build capacity for researchers to apply health equity are through mentoring relationships. This is an area where our Center works in partnership with other initiatives at Wash U to support the training and mentorship of early career researchers and those who are mid-career and shifting to a focus on implementation science. For example, the Center has a T32 and an R25 focused on training and mentorship to build pathways and opportunities for diverse scholars in implementation science. These grants allow Center members to receive training and mentoring specifically focused on implementation science and health equity. The opportunities for learning about health equity through training and webinars are typically offered through other areas of Washington University (e.g., the Institute for Public Health’s Center for Dissemination and Implementation) or the broader implementation science field although our Center supports those initiatives*.”

Several Centers also described the development and dissemination of tools and resources with the explicit goal of advancing health equity in implementation science for the ISC^3^ program and the broader field.

Example 4.2 UW OPTICC: “*One of UW OPTICC’s signature methods is a graphical tool called causal pathway diagrams (CPDs). Each of our pilot projects uses CPDs to depict how implementation strategies work in the context in which they will be implemented. Our Center helped a trainee develop CPDs for four known barriers, which helped operationalize study content, framing, graphics that the trainee vetted with end users in rapid prototyping focus group sessions to ensure the materials were patient-centered. We oversampled minoritized populations in our end user prototyping sessions to surface different barriers that might be experienced by different groups and tailor our materials accordingly*.”

### Domain 5: Engaging and Including Underrepresented Scholars in Implementation Science

Centers had variable levels of institutional and research resources and efforts dedicated to engaging, training, including, supporting, and retaining scholars in implementation science from underrepresented populations (as defined in the context of the US Biomedical, Clinical, Behavioral, and Social Sciences workforce [34]). Many Centers reported wanting to develop and apply best practices to advance impact in this critical area. Several Centers reported leveraging external resources to engage or support the diversity and inclusion of scholars within the Centers. For example, some Centers were leveraging broader department, school, and/or University resources and environments, such as existing T32s and other existing health equity training programs, to help engage and support diverse scholars who were interested in implementation science. Centers also reported leveraging diversity and/or administrative supplements to ISC^3^ awards to provide support for scholars and faculty who are underrepresented groups (as defined by NIH):

Example 5.1 iDAPT: *“Career training pathways in the iDAPT Center can begin at varying stages: doctoral, post-doctoral, early career. At Wake Forest School of Medicine, we collaborate with the T32 NCI-funded cancer training program to identify post-doctoral fellows who are interested in participating in the iDAPT Center activities. We also offer a ’scholar in residence’ program, which includes an invitation to diverse scholars to engage with the Department and Center activities. At UMass Chan, we collaborate with the T32 NCI-funded PRACCTIS training program, the NIH K12 Cardiopulmonary Implementation Science Scholars Program, and the UMass Chan Cancer Center’s Program in Cancer Population Health Sciences to identify post-doctoral fellows and early-stage investigators interested in participating in iDAPT Center activities. Our faculty hold joint appointments at the Medical School and the Graduate School of Biomedical Sciences, allowing us to formally provide mentorship for pre-doctoral candidates, an important stage of our ‘pipeline’.”*

Centers noted the challenges and lessons learned from their efforts thus far, including short-term and circumscribed funding opportunities, the isolated nature of remote work and challenges building a sense of academic community amidst COVID-19, and broadening the typical channels used to advertise post-doctoral training opportunities.

Example 5.2 UW OPTICC: “*Our center prioritizes supporting diverse scholars through funding and training opportunities. We sought and obtained an NCI Diversity Supplement for our Center team. While it is a strategy for providing deep support with funding to students and junior investigators, it will have limited reach at each Center as there are limitations in the number of supplement grants for diverse trainees. However, Diversity Supplements are underutilized and can really help support racially/ethnically diverse and other underrepresented scholars ranging from students to investigators*.”

## Discussion

This report provides applied examples, shares challenges, and highlights opportunities to integrate a focus on health equity in implementation science from a national network of implementation science centers. Centers reported a wide range of approaches in applying and operationalizing health equity in research and capacity-building efforts in the early stages of center development (years 1–3 of a five-year initiative). Potential opportunities to explicitly focus on health equity in implementation science in research networks identified from the survey results included: (a) assess and/or measure the impact on health equity-related outcomes in the context of implementation science; (b) promote the use of TMFs, measures, and evaluation metrics that include a focus on health equity to allow for comparisons across contexts and populations; (c) prioritize capacity-building efforts in implementation science that engage, include and support investigators across a range of career levels and diverse backgrounds to reflect the diversity of communities impacted by cancer; and (d) share and disseminate both successes as well as challenges and lessons learned from focusing on health equity in implementation science. In the following sections, we provide recommendations for other investigators’ and centers’ efforts to build capacity and infrastructure in this area.

### Identify Effective Approaches for Engaging Community Partners in Health Equity-Focused Implementation Science

Engaging community partners in the earliest stages of implementation efforts and on an ongoing basis throughout implementation is a best practice for centering the voices of those most impacted and elevating a more grounded understanding of equity [12,17]. However, with some exceptions [20,21,35], there have been limited empirical investigations of optimal approaches and strategies for engaging community partners in implementation science focused on health equity. Community-based participatory research is a well-established method for engaging community members and partners to increase health equity [36] and can be used in an implementation science context. A unique aspect of community engagement in implementation science is the importance of eliciting community members’ perspectives on the value and demand for the interventions and programs being implemented as well as seeking the input of community members’ and other key partners (e.g., providers, practitioners, administrators) on the barriers and facilitators to equitable implementation. To advance a focus on health equity in implementation science, the field will need to identify approaches and strategies for effectively engaging community partners at various levels, from organizational leaders to community residents, and in different types of engagement activities (e.g., sharing information, consultation, co-designing implementation strategies). *The Conceptual Model to Advance Health Equity through Transformed Systems*, led by the National Academies of Medicine Organizing Committee for Assessing Meaningful Community Engagement in Health & Health Care Programs & Policies, is a resource for community-engaged, effective, and evidence-based tools to those who want to measure engagement to ensure that it is meaningful and impactful, emphasizing equity as a critical input, process, and outcome [37]. In addition, learning from other disciplines (e.g., sociology, anthropology) that have successfully engaged community partners in research and scholarship focused on advancing health equity could inform efforts to do similar work in the field of implementation science.

Relevant community engagement constructs and processes previously identified in implementation science, including communication, partnership exchange, community capacity-building, leadership, and collaboration [38], may be useful in these efforts. An important next step is to begin to refine and specify these broader community engagement constructs and processes in implementation science TMFs that include a focus on health equity. In addition, investigators working in research networks can build on examples of implementation research specifically focused on engaging community partners in meaningful ways with diverse perspectives, experiences, and expertise in making decisions about research priorities and data focused on health equity [39].

### Promote the Use of TMFs, Measures, and Metrics that Include a Focus on Health Equity

Another opportunity to advance a focus on health equity in implementation science is to promote the use of implementation TMFs, measures, and evaluation metrics focused on health equity [4,6,13]. Aligning health equity constructs with measures and metrics used in implementation science [40], can help investigators identify relevant constructs and measures for their particular context, or address or frame research questions. In addition, integrating and operationalizing these health equity constructs in TMFs consistently in a range of studies across varying settings and populations in research networks and reporting this work will help the field replicate research and develop cohesion in identifying relevant determinants and outcomes. Key questions in operationalizing, measuring, and evaluating health equity include:

- *Which health equity domains do clinical, community, and public health partners prioritize across a diverse range of settings?*
- *Which TMFs developed to address health equity from outside implementation science are being leveraged with successful outcomes?*
- *What are ways to determine when implementation efforts and/or strategies have equitable impact?*

### Education, Training, and Mentored Research on Health Equity-Focused Implementation Science

For research center networks, we recommend providing education, training, and mentored research opportunities for investigators interested in focusing on health equity in implementation science within the institution and across the network. To date, implementation scientists have found innovative ways to deliver and scale educational materials, training, and resources to meet a wide range of learning needs, including online modules with readings, mentored experiential learning, and video libraries [41]. Similar approaches could be applied to health equity principles and considerations to provide a solid foundation in these topics to the implementation science workforce. In addition to these more traditional approaches, there is ample opportunity for innovation in the process by which we provide education and training for focusing on health equity in implementation science. For example, public health scholars have recommended using creative expression such as music, poems, and painting to help people think creatively about efforts to eliminate or mitigate the health effects of racism [42]. We recommend investigators consider piloting these innovative approaches in their own networks. Funding opportunities should be developed to support capacity-building activities related to education, training, and mentoring in health equity-focused implementation science similar to other training initiatives [43,44].

### Increase Diversity and Inclusion of Scholars who are Underrepresented in the Implementation Science Workforce

Another significant opportunity to advance health equity in implementation science is to engage, include, and support the professional and research career growth of scholars underrepresented in the scientific workforce. Such efforts will help to increase the breadth and depth of research to reflect broader social contemporary issues and injustices [45]. Promoting engagement, inclusion, and support of underrepresented scholars in implementation science will require commitment by institutional leadership, national organizations, and government to prioritize and sustain diversity, equity, inclusion, and accessibility. Building synergies in capacity building by setting strategic priorities and developing cross-center policies within research networks to form a group of national and local mentors can leverage existing resources and talent, and help foster a culture of supporting and including underrepresented scholars in implementation science.

### Disseminate Challenges and Lessons Learned Conducting Health equity-focused Implementation Science

Finally, sharing challenges and lessons learned in developing research and capacity building that focuses on health equity will help identify where change and resources are needed [46,47]. Centers reported practical challenges from their efforts thus far, including short-term and circumscribed funding opportunities, the isolated nature of remote work/telework (during the COVID-19 Pandemic), challenges building a sense of academic community amidst COVID-19 global pandemic, and identifying successful recruitment and retention strategies for building a diverse workplace. Future directions include sharing lessons learned about these challenges and effective strategies to manage them (e.g., collaborating with university hiring managers to strengthen diversity & inclusion efforts).

Additional challenges of doing this work include the unintended exacerbations of inequities of implementation research. Investigator willingness to disclose and share these experiences with others can help mitigate such consequences in the future. Incorporating theories that explicitly focus on power, social inequities, and structural racism affecting communities and individuals [27,48] can bring these issues to light. In addition, recognizing and addressing the challenges of historic and ongoing racism and implicit bias in academia [49] are critically important to effective capacity-building efforts in health equity and implementation science. The ISC^3^ network has a cross-center evaluation work group that conducts annual evaluations of each center and a bi-annual social network analysis [50]. These evaluation efforts include a logic model that tracks health equity-related inputs, processes, and outcomes, and the social network analysis maps collaborations within and across centers by race/ethnicity, gender, career level, and discipline. These evaluation efforts are essential for continuously documenting challenges and lessons learned; identifying promising practices and approaches; sharing opportunities for collaboration and highlighting new ideas for integrating health equity across the domains identified in the current report.

There is enormous potential for large research centers, research networks, and hubs across the U.S. (beyond the ISC^3^) to work together to accelerate the growth of health equity-focused implementation science. The Clinical and Translational Science Awards (CTSA) Program supports a national network of medical research institutions (i.e., hubs) that work together to accelerate the translational research process. One goal of the CTSA program is to disseminate innovative research programs and partnerships across institutions and communities to address health inequities and increase the reach of translational science. In addition, the Prevention Research Centers of the Centers for Disease Control and Prevention are a national network of research centers that study how communities can reduce the risk for chronic disease, with a focus on solutions that can be applied widely in marginalized populations. Together, networks with similar emphasis on implementation science and advancement of health equity may consider partnering to facilitate the large-scale dissemination of lessons learned, engage in collaborative research and training opportunities, and share effective engagement approaches. Initial ideas for innovations include hosting virtual and in-person meetings and events highlighting health equity-focused implementation methods and projects that provide explicit opportunities for community member engagement and input; such events could provide innovative research training programs that bring together mentorship, educational, and research opportunities across networks of Centers and Hubs (e.g., short-term research residences in health equity-focused implementation science at CTSAs, PRCs, and ISC^3^s). To take action on these ideas, partners may consider incentives and greater value and culture for collaborative achievements and co-learning, open access, and shared resources, across funded networks of health equity-focused Centers and Hubs.

### Limitations

The results of this survey are from a network of seven Centers in the NCI-funded ISC^3^ program engaged in early work to develop research and build capacity for integrating health equity in implementation science. Respondents were instructed to provide illustrative case examples related to domains that were most relevant to their respective centers. The case examples presented in this report are not necessarily a comprehensive overview of health equity-focused activities across the centers, nor do they highlight all of the challenges of doing this work well. Also, while we acknowledge that majority of HETF members and co-authors on this manuscript are white female academics, there is representation in ISC^3^ from other groups underrepresented in science. Similar to capacity-building efforts reported by the ISC^3^ Centers, the ISC^3^ HETF actively seeks to increase engagement and inclusion of individuals underrepresented in the implementation science workforce to increase diversity of members’ perspectives.

## Conclusions

ISC^3^ centers reported a wide range of approaches in applying health equity in research and capacity-building efforts. Recommendations for advancing opportunities to focus on health equity in implementation science include identifying effective approaches for engaging community partners in health equity-focused implementation science; harmonizing implementation theories, models, and frameworks, measures, and metrics that include a focus on health equity; education, training, and mentored research focused on health equity in implementation science; increasing diversity and inclusion of scholars who are underrepresented in the implementation science workforce; and disseminating challenges, potential solutions, and lessons learned conducting health equity-focused implementation science. Dedicated leadership supported by strategic resources can help investigators pursue these research and capacity-building opportunities to advance health equity in implementation science.

## Supporting information

Aschbrenner et al. supplementary material Aschbrenner et al. supplementary material

## Supplementary material

The supplementary material for this article can be found at https://doi.org/10.1017/cts.2023.638.
