# Supplementary material for: Integrating a focus on health equity in implementation science: Case examples from the national cancer institute’s implementation science in cancer control centers (ISC3) network
Source: J Clin Transl Sci. 2023 Sep 29;7(1):e226. doi: 10.1017/cts.2023.638 (PMC10643915; doi:10.1017/cts.2023.638)
Supplement: Aschbrenner et al. supplementary material [file S2059866123006386sup001.docx]

**Supplementary File 1.** Survey Responses to Five Health Equity and Implementation Science Domains

| **Domain** | **ISC3** | **Case Example** |
| --- | --- | --- |
| 1) Community engagement in health equity-focused implementation research | WU-ISCCC | “*We have engaged stakeholders to guide the prioritization of research areas for and the review of pilot grant proposals within our center. The Implementation Laboratory (referred to as the Innovation Incubator within our Center) has been engaging with a wide array of community members across the 82-county catchment region of the Siteman Cancer Center and representatives from key organizations working to promote health in the region. Members of the Incubator come from both urban and rural settings and represent a range of socioeconomic, racial and ethnic groups. We have a diverse group of organizations represented in the Incubator that work across the cancer prevention and control spectrum, including those focused on direct service/program delivery, capacity building, and advocacy*.” |
|  | Penn ISC3 | “*Penn ISC3’s Implementation Laboratory (iLab) comprises of 12 sites and approximately 500 clinicians across the Abramson Cancer Center (ACC). As the ecosystem in which to conduct the Center’s implementation work, iLab created the foundation and hub for the launch and successful run of the pilot projects; to conduct bi-directional engagement with various stakeholders; securely manage data, and perform practice surveillance….during the nudge design process, clinicians representing each ACC site participating in the pilot projects were invited to partake in a Penn ISC3 clinician-based focus group to provide feedback on the first two signature pilot projects, including communication preferences, current challenges encountered, clinician nudge layout and content, and patient nudge layout and content. The ACC Patient and Family Advisory (PFAC) comprises of a group of patients, caregivers, and social workers from the ACC with experienced knowledge of cancer treatment. We engaged the PFAC to garner feedback on both pilot studies, including nudge content, communication preferences, nudge delivery timing, and layout. Stakeholders identified various social determinants of health as possible barriers in accessing the interventions, including technological barriers and choice of communication platform. Stakeholders provided invaluable feedback and recommendations to address their concerns*.” |
|  | UW OPTICC | “*One of our pilot projects focused on developing a rideshare intervention for colonoscopy completion for patients served by a safety net health system. The goal of this project was to optimize rideshare non-emergency medical transportation (NEMT) for colonoscopy, which requires procedural sedation. The health system was engaged in initiatives to increase colon cancer screening and had noted suboptimal colonoscopy follow-up for positive FIT tests. The project team learned from patients that transportation was a key barrier to completing colonoscopy. Transportation (or lack of it) is a social determinant of health and is frequently cited as limited access to care among underserved communities. For colonoscopy, lack of transportation and requiring an escort home (due to procedural sedation) negatively impact health equity. Other stakeholders that were initially identified included key members of health system staff and leadership as well as rideshare companies. Conversations with these stakeholder groups identified other key stakeholders that participated in later conversations. Ultimately, 34 stakeholders participated in informal conversations and/or group meetings. The study team used story boards as a creative way to engage stakeholders about benefits, barriers, and facilitators to using rideshare NEMT for colonoscopy patients. Stakeholder engagement and input led to the development of a rideshare NEMT strategy that is optimized to meet various stakeholders’ needs and is currently undergoing pilot testing*.” |
|  | Harvard ISCCE | “*In the first round of ISCCCE’s cancer prevention and control pilots, the I-Lab played a significant role in engaging Community Health Center (CHC) stakeholders by providing support and technical assistance as they implemented a paired cancer screening and social determinants of health pilot. This work has included building capacity and engagement with community members and/or community-based organizations (CBOs). As an example*, *I-Lab staff worked directly with CHC stakeholders throughout the implementation process, starting with a needs assessment with CHC teams to learn about their response to COVID, how they were addressing social determinants of health and learning about their current FIT [colorectal cancer screening] test workflows. In the guided adaptation phase of the methods pilot, the I-Lab provided implementation facilitation to CHCs to conduct data comparisons of outreach and use of the intervention. This included individuals reached for colorectal cancer screening and those who completed screening through the pilot implementation phase with the goal of identifying population groups not being reached or screened with current workflows (for example, by language or race) in order to modify strategies to reach those groups*.” |
|  | BRIDGE-C2 | “*The I-Lab at the BRIDGE-C2 Center formalized collaboration with OCHIN’s* ***Health Resources and Services Administration (HRSA)*** *-funded Health-Centered Controlled Network (HCCN). [OCHIN is a non-profit healthcare innovation center]…The HCCN Network is focused on quality improvement (QI) for federally qualified health centers (FQHCs) and look-alikes to aid in health equity. These connections provide the BRIDGE-C2 Center with strategic alignment around knowledge of QI initiatives (e.g., interactive colorectal cancer screening decision tree) and electronic health record (EHR) enhancements (e.g., colorectal cancer and cervical cancer screening Health Maintenance Topics). These connections also allow the Center to leverage resources across teams for evaluation of specific interventions, survey activities and to access a cohort of clinics for deeper investigation (e.g., qualitative interviewing), and understand community needs around health equity*. *For example, in May 2020, HCCN and BRIDGE-C2 co-designed an evaluation tool and conducted interviews with OCHIN staff about their technical assistance and QI efforts and the impacts that COVID-19 had on their direct work with community health centers. The I-Lab connects with HCCN and clinical team members through monthly meetings. This bidirectional support and community building at the leadership level offers multi-level engagement with downstream impacts particularly around health equity.”* |
| 2) Emphasizing health equity in implementation theories, models and frameworks | WU-ISCCC | *“In a recently-funded project, a researcher from the Center will use the Health Equity Implementation Framework (1) to guide work to quantify and characterize the direct costs, including the financial burden from insurance related fees (e.g., co-pays, co-insurance) and indirect costs, including the unforeseen costs (e.g., loss of work, absenteeism, presenteeism), to support the implementation of shared decision making among Black men with prostate cancer as they weigh the tradeoffs between treatment options.”* |
|  | Penn ISC3 | “*Our approach towards operationalizing health equity related measures into the Penn ISC3 corpus of work is broadly informed by several implementation science frameworks. The Consolidated Framework for Implementation Research (CFIR)(2) determinants framework broadly informs our focus on both the Outer and Inner Contextual levels that shape health inequities and inequitable implementation. Additionally, we will expand upon potential equity-related determinants that shape these inequities by examining contextual, provider, and patient-level factors that might explain these findings (e.g. why were nudges inequitably adopted/effective across different racial/ethnic or socioeconomic groups among patients? Why might providers differentially refer patients?), informed by The Health Equity Implementation Framework (1). Specifically, we will oversample patients by race and income to explore these differences. Additionally, in the signature pilot projects, we are integrating CFIR with the RE-AIM Extension for Equity and Sustainability (3) to inform selection of our implementation indicators and outcomes and tracking equitable/inequitable adoption, reach, and implementation in relation to behavioral nudges, as well as potential differences in patient perceptions of acceptability and appropriateness of implementation strategies (by income and race/ethnicity). Challenges here have been having sufficient variation in patient/setting characteristics to examine some of these differences; additionally, it can be challenging to adequately capture some of the contextual factors that may be at play (e.g. provider or system biases*).” |
|  | Harvard ISCCCE | *“As part of the pilot, “Clinical‒Community Partnerships for Cancer Prevention Equity”, we included questions to develop a greater understanding of how aspects of the outer setting were impacting community health centers and community-based organizations throughout the state. This approach to improving understanding of the outer setting was delineated first in the equity-focused elements we added to the CFIR framework(2) we proposed to guide our center in our proposal. The extension of the outer setting in these qualitative interviews included the COVID-19 context as well as the heightened attention around structural racism and police violence. COVID-19 contextual questions were adapted from an interview guide shared by colleagues from Washington University via the ISC3 measures working group. The core questions focused on equity were: 1) How have coronavirus restrictions affected pre-existing disparities in your areas? What groups have been most affected? Where are additional supportive actions most needed? Impacts on people of color, children, elderly, food security, jobs? and 2) The issues of racism and police violence have also recently become public health issues at the forefront of the American consciousness. How has this national attention impacted your agency? Have additional resources (e.g., financial, personnel) been made available to you in light of recent events? What do those look like?”* |
|  | BRIDGE-C2 | “*The Practice Change Model (PCM)(4) identifies factors (and their interdependences) that shape primary care practices’ ability to change and influence intervention uptake. The PCM differentiates motivation (energy for change) and capacity or resources for change (capability). It emphasizes the importance of interdependencies that manifest among the contextual and environmental factors influencing intervention effectiveness. In other words, the PCM identifies multilevel structural determinants that impact practices and the care they provide. This model helps highlight potential actions to increase health equity at patient-, provider-, clinic- levels. BRIDGE-C2 pilots are guided by this model. Our Research Program’s process to select pilots integrates with the Implementation Laboratory units to rapidly assess community health centers’ priorities, capacity and quality indicators; our initial methods work is focused on setting the stage to more precisely identify the factors that shape and predict appropriate strategies and what level of implementation support is needed by a specific practice (or group of practices with similar characteristics) prior to and during active implementation. Practice change dynamics will inform the current and subsequent research to be completed in the BRIDGE-C2 Center. Pilots are encouraged to fit their projects into this framework early, ideally during pre-pilot planning. This allows us to identify health equity determinants to guide implementation mechanisms and equity-related measurable outcomes*.” |
|  | Colorado ISC3 | “*Our center is leading work on identifying a) health equity constructs in D&I frameworks, b) D&I constructs in selected leading health equity frameworks; and c) providing case examples and guidelines for combining and adapting these frameworks. This work is being conducted as a major expansion of our dissemination-implementation.org website tool and funded by an NCI supplement*.” |
|  | UW OPTICC | “*Investigators in OPTICC have developed pragmatic, brief implementation measures that are acceptable and relevant to a wide variety of stakeholders. The causal pathway diagramming (CPD) approach also helps practice and research partners create their own mini-theory of how an intervention will work in their specific context. They can use CPDs to diagram how their specific context and the needs of their specific populations interact with (or may lead to barriers and facilitators) implementation of a specific evidence-based intervention*.” |
| 3) Evaluating health equity in application of processes and outcomes | Colorado ISC3 | ***“****The aim of the pilot is to conduct a feasibility trial of the implementation of a patient-centered shared decision making and lung cancer screening (LCS) strategy in rural primary care clinics in Colorado. This intervention package includes a formal shared decision making (SDM) process and smoking cessation support for rural primary care clinics that is aligned with Center for Medicare and Medicaid Services (CMS) criteria for LCS. We are using the PRISM (5) and RE-AIM frameworks (3, 6) to guide our evaluation- as well as planning and implementation. Importantly we are focusing on issues of both: a) representation (who is involved in planning, execution and evaluation at each of the RE-AIM ‘steps’); and b) representativeness (or equity) of outcomes on each RE-AIM dimension to evaluate the cumulative or ‘Cascade’ effect of potential inequities at each point. Trying to comprehensively assess all potential equity-related factors, and especially context is an overwhelming task. For example, on each RE-AIM dimension we are assessing representativeness but on what specific factors and how many factors is a challenge. These decisions are based on both the literature and on factors from our experience that are most likely to be strongly related to outcomes, as it is impossible to address everything.”* |
|  | Wake Forest iDAPT | “*One of our investigators is developing a “Usable Measure for Digital Divide in the Clinical Setting” as a methods pilot study. Digital tools can improve effectiveness and reduce healthcare barriers. However, to fully realize their potential (to broaden reach to all populations), implementation strategies must address the digital divide challenge. The digital divide for health-related technology may be a result of various factors. At the person level, it can include motivation, personality traits (e.g., openness, extraversion, conscientiousness), and digital skills. Social determinants of health also impact the digital divide. At the population-level, this understanding will allow implementers to develop the appropriate strategies to ameliorate or eliminate disparities. Understanding these factors and their interactions is helpful for clinical implementers to address the digital divide. Our first goal is to identify electronic health record “markers” for measures for the digital divide. These markers can be* *augmented with questions (as deemed appropriate) to allow clinical implementations to better measure and address the value of digital tools during clinical implementation*.” |
|  | BRIDGE-C2 | “*The BRIDGE-C2 Cervical Cancer Screening Tool pilot aims to evaluate adoption of an EHR-based implementation strategy [CC tool], which was designed by OCHIN to assist clinical teams with providing evidence-based cervical cancer screening interventions and implemented in our Implementation Laboratory of community health centers. The pilot also examined multilevel health equity determinants that impact the adoption of this tool. The Implementation Laboratory updated the EHR tool to reflect new guidelines, which the pilot will measure. An important component of this pilot was using results from studying uptake of earlier versions of the tool to guide user-centered design process with community health center clinicians to influence the changes to the tool. The user-centered design process yielded important health equity factors and outcomes that the research team then incorporated into our evaluation process. For example, clinicians noted needing a tool to help them identify patients who had missed cervical cancer screening appointments or had delayed follow-ups after a positive screen – two important outcomes informing the tool and critical for health equity. Clinicians emphasized the importance of understanding how to reach patients to complete the screening process and to understand which patients were most likely to need this outreach. The pilot team incorporated completion of the cervical cancer screening process (including referrals, follow-up care, and patient-provider communication) into our evaluation of the adoption and sustainability of the tool*.” |
|  | Harvard ISCCCE | “*Our Methods Unit has conducted formative research on the development of a Stakeholder and Equity Data-Driven Implementation (SEDDI) process(7) where healthcare partners use data to identify patient groups that experience gaps in service access and benefits from EBIs and rapidly adapt EBIs to address gaps in care. We applied SEDDI in a pilot hybrid implementation-effectiveness trial of a paired colorectal cancer (CRC) and social needs screening intervention at four federally qualified community health centers (CHCs). An external facilitator partnered with CHC implementation teams over eight-months supporting a base implementation phase followed by the SEDDI phase delivered in bi-weekly or monthly sessions. Preliminary evaluation of SEDDI involved convergent mixed methods with surveys and focus groups. CHCs were able to obtain and use clinic data to identify gaps in outreach for the paired screening intervention by race/ethnicity, gender, age and language and make adaptations to improve outreach strategies and tailor the EBI. Adaptations included cultural, linguistic, and health literacy tailoring. SEDDI was highly acceptable and feasible to implement. CHC teams liked the structured facilitation process and found it useful to use clinic data to identify and prioritize gaps in outreach. None of the four CHCs completed rapid cycle testing due to time constraints in the project period*. *Our team is collaborating with investigators at Wake Forest* iDAPT *to use human centered design principles in partnership with CHC stakeholders to further improve SEDD’s usability by CHC staff in CHC environments.”* |
|  | UW OPTICC | *One of our new pilot projects focuses on optimizing practice facilitation as a strategy for increasing colon cancer screening in Federally Qualified Health Centers (FQHCs). This study will enroll 8 FQHCs, which are health systems serving primarily low income and underinsured communities. Six FQHCs will receive standard practice facilitation through an existing state-funded CRC screening program and two Control FQHCs will receive standard practice facilitation and the intervention FQHCs will receive optimized practice facilitation that will be tailored to their highest-priority barriers to implementing evidence-based interventions that increase colon cancer screening. We will use the Adapting Strategies to Promote Implementation Reach and Equity (ASPIRE) framework(8) to guide our practice facilitation optimization, while applying at least two OPTICC optimization methods. We will examine both implementation of the evidence-based interventions each FQHC selects as well as change in screening rates for patients. We will examine change in screening rates by patient ethnicity (Latino/not Latino) and primary language (Spanish/English).”* |
|  | Penn ISC3 | “*To illustrate how we have operationalized equity TMFs into Penn ISC3’s work, we describe it for our signature pilot project studying the effect of behavioral economic implementation strategies for increasing referrals to tobacco use treatment services (TUTS). First, we have measured inequities at baseline to TUTS within the prioritized three populations: race/ethnicity, socioeconomic status, and rural/urban setting. Second, we are currently monitoring if inequities (in health or implementation) might widen or appear during the implementation phase within the three prioritized populations. Third, we plan to analyze inequities that might widen or appear after the intervention has been fully implemented and the follow-up period has been completed. Fourth, we are currently oversampling for subgroups within our three populations of interest for qualitative interviews. Among patients, questions will probe participants about reactions to the nudge and referral and, in line with our health equity lens, questions include those about social and structural factors that may contribute to health inequities such as experiences of discrimination, medical mistrust, perceived health care access, and language barriers. We will take a similar approach in our signature pilot project studying serious illness conversations. As described in the question above, we are using Proctor framework to assess perceptions of strategies (acceptability, appropriateness) and RE-AIM extension to track health and implementation inequities. For both research studies (and thus the patient interviews), we are geocoding individual patients and mapping to neighborhood/census tract SDOH. We will explore moderating effects by neighborhood-level factors (e.g., % living under the poverty line; education; Yost index) in trial analysis. Additionally, for patients where we also have either a) EMR social risk factors or b) survey responses (from MP2) assessing individual SDOH (e.g., education), we will compare and contrast the relationship between individual and neighborhood-level SDOH on effectiveness of nudges and clinical outcomes*.” |
|  | WU-ISCCC | “*As part of ongoing efforts within our Practice Surveillance Unit, we are creating Community Snapshots and a data dashboard to disseminate local data on social determinants of health and cancer care [https://implementationscience.wustl.edu/home/innovation-incubator/community-snapshots] to our clinic and community partners. These tools were developed to address a need for local data to inform grants, advocacy, and community- and clinic-driven implementation efforts expressed by our partners. Following partner recommendations, the Snapshots also include a personal story, implementation challenges related to the featured social determinant of health, and resources (e.g., services, research experts). The first snapshot was focused on transportation – its role in cancer care and how implementation science can work to address this often barrier to cancer care. In the second aim of our outer context supplement, we will expand the range of social determinants of health included in the Snapshots and support an evaluation of the extent to which the Community Snapshots successfully serve their intended audiences, across the following dimensions: reach, accessibility, relevance, and impact. We have developed survey questions to access characteristics of end-users (reach), perceived ease of access, clarity, delivery, and format (accessibility), alignment with community concerns and priorities, personal and organizational goals (relevance), and knowledge about content and use/intention to use content (impact). Surveys will be distributed via email to our Implementation Laboratory membership and by clinical and community partners. The goal of the qualitative interviews is to deepen our understanding of how well the Snapshots served their audiences and/or what can be improved*.” |
| 4) Building capacity for focusing on health equity in Implementation science | Wake Forest iDAPT | “*The NCI Community Oncology Research Program (NCORP) is one of iDAPTs iLABS. As both partner and stakeholder, we are building capacity for health equity in implementation research with cancer care delivery researchers (CCDR) at NCORP community sites. We do so in two ways: formal workshops/training and 1-on-1 in-depth consultation/mentoring on research projects*.” |
|  | BRIDGE-C2 | “*The BRIDGE-C2 Center established a Request For pilot study Applications (RFA) to fund rapid-cycle small pilots that have a health equity focus and are aligned with the BRIDGE-C2 Center’s Grand Challenge – advancing implementation science to improve cancer screening and prevention in underserved populations. BRIDGE-C2 gives high priority to funding researchers across career stages, especially junior faculty, multi-disciplinary teams (i.e. clinical & non-clinical members), and learners (e.g. students, post-doctoral, K scholars). We knew that these target grantees may not have an advanced understanding of conducting and managing real-world implementation science research pilots. Therefore, we established a robust mentorship strategy, both implementation science experts as well as project management and budget management experts. We offered four “office hours” once the RFA was posted, to create highly accessible pathways for potential applicants to learn more and seek feedback on their research ideas. We offer grantees opportunities to present their work in multiple types of venues for career development and feedback in addition to accessing Center trainings, seminars, Grand Rounds presentations and our multi-disciplinary implementation science workgroup. We designed the pilot RFA process to build capacity for applying health equity in implementation science research careers.”* |
|  | Harvard ISCCCE | “*We try to take advantage of every opportunity to build the research capacity of our community partners. For example, our publication policy requires that all ISCCCE papers include CHC partners as co-authors. We have developed a set of roles that are appropriate to their time availability but also bring significant value to our data interpretation and manuscript development. We have supported opportunities for CHC partners who have strong research interests to participate as co-investigators and to gain more advanced research training*.” |
|  | UW OPTICC | “*One of UW OPTICC’s signature methods is a graphical tool called causal pathway diagrams (CPDs). Each of our pilot projects uses CPDs to depict how implementation strategies work in the context in which they will be implemented. Our Research Program Core helped a trainee develop CPDs for four known barriers, which helped operationalize study content, framing, graphics that the trainee vetted with end users in rapid prototyping focus group sessions to ensure the materials were patient-centered. We oversampled minoritized populations in our end user prototyping sessions to surface different barriers that might be experienced by different groups and tailor our materials accordingly.”* |
|  | Penn ISC3 | “*By partnering with the Penn Implementation Science Center (PISCE@LDI), the Penn Center for Cancer Care Innovation (PC3i), other centers at the university, and the 65+ T32s, several of the trainees of Penn ISC3 investigators have also received NIH career development awards or other grants for their work, and many of these grants include aims associated with health equity. Still, we acknowledge that there is much more that we can do to promote training at the intersection of health equity and implementation science. While we are still in the early investment phase in this area, Penn ISC3 hopes to set up more formal training in this space, leveraging our partnership with an international expert, [name removed], who is part of our Penn ISC3. One idea is to add a track to our annual Penn Implementation Science Institute, which trains approximately ~100 people annually, focused on the intersection of health equity and implementation science; or adding a day focused on this matter. We ask all of our speakers to attend to health equity in their presentations, but this would represent an opportunity to create more focused training.*” |
|  | WU-ISCCC | “*In our Center, the main ways that we build capacity for researchers to apply health equity are through mentoring relationships. This is an area where our Center works in partnership with other initiatives at Wash U to support the training and mentorship of early career researchers and those who are mid-career and shifting to a focus on implementation science. For example, the Center has a T32 and an R25 focused on training and mentorship to build pathways and opportunities for diverse scholars in implementation science. These grants allow Center members to receive training and mentoring specifically focused on implementation science and health equity. The opportunities for learning about health equity through training and webinars are typically offered through other areas of Washington University (e.g., the Institute for Public Health’s Center for Dissemination and Implementation) or the broader implementation science field although our Center supports those initiatives*.” |
| 5) Engaging and including underrepresented scholars in implementation science | Wake Forest iDAPT | *“Career training pathways in the iDAPT Center can begin at varying stages: doctoral, post-doctoral, early career. At Wake Forest School of Medicine, we collaborate with the T32 NCI-funded cancer training program to identify post-doctoral fellows who are interested in participating in the iDAPT Center activities. We also offer a “scholar in residence” program, which includes an invitation to diverse scholars to engage with the Department and Center activities. At UMass-Chan, we collaborate with the T32 NCI-funded PRACCTIS training program, the NIH K12 Cardiopulmonary Implementation Science Scholars Program, and the UMass Chan Cancer Center’s Program in Cancer Population Health Sciences to identify post-doctoral fellows and early-stage investigators interested in participating in iDAPT Center activities. Our faculty hold joint appointments at the Medical School and the Graduate School of Biomedical Sciences, allowing us to formally provide mentorship for pre-doctoral candidates, an important stage of our ‘pipeline.”* |
|  | UW OPTICC | “*Our center prioritizes supporting diverse scholars through funding and training opportunities. We sought and obtained an NCI Diversity Supplement for our Center team. While it is a strategy for providing deep support with funding to students and junior investigators, it will have limited reach at each Center as there are limitations in the number of supplement grants for diverse trainees. However, Diversity Supplements are underutilized and can really help support racially/ethnically diverse and other underrepresented scholars ranging from students to investigators*.” |
|  | Penn ISC3 | “*Investigators from the Penn ISC3 team devote substantial time to mentorship, particularly for scholars and trainees from historically underrepresented backgrounds as defined by the National Institutes of Health. In a recent survey, core team members indicated that they provide mentorship for more than 50 early-career investigators to date, many of whom come from backgrounds historically underrepresented in the sciences. Notably, there was a significant range in both mentees’ stage of career development (undergraduate and graduate students to early-career clinicians and faculty) and research areas (digital health and artificial intelligence to human decision-making). Still, we acknowledge that there is much more that we can do to promote the recruitment, training, and development of historically underrepresented scholars. While we are still in the early investment phase in this area, Penn ISC3 hopes to set up more formal training programs for people who come from historically underrepresented groups and are interested in implementation science or cancer care. By partnering with the Penn Implementation Science Center (PISCE@LDI) and other centers at the university, we hope to foster an even more inclusive community that promotes diversity in implementation science and cancer care at Penn*.” |
|  | Harvard ISCCCE | *“The I-Lab provides opportunities to trainees and students of color to explore the field of implementation science and community health. The I-Lab hosted a post-baccalaureate pre-med student through Health Career Connections during Summer 2020. The intern supported the team’s capacity building work and provided an opportunity for her to explore implementation science topics and cancer prevention research. In Summer 2021, The I-Lab hosted a bachelor’s level student through Dana Farber Cancer Institute’s CURE program that focused on developing a research project and presentation related to pre and post COVID cancer related risk factors. The I-Lab is also working with an undergraduate level student to create a multilevel policy dataset to examine the policies affecting the implementation of COVID testing services in community health centers in our RADx-UP supplement.”* |
|  | WU-ISCCC | “*We have a variety of trainees and faculty in our Center, including Master’s and Doctoral students, post-doctoral trainees, research staff, and faculty who are early-, mid-, and late-career as well as those who have differing levels of expertise with implementation or health equity research. The members of our Center represent a diversity of racial and ethnic backgrounds, first-generation students and faculty, those from highly urban or rural areas, LGBTQIA+ individuals, and those with physical disabilities. In our Center, it is important that our scholars represent these categories and other historically (and currently) marginalized groups to ensure that the work produced as part of Center initiatives and pilot grants is in line with the health equity-related goals of the Center. Challenges and lessons-learned from the equitable hiring, retention, and mentorship of our post-doctoral scholars are somewhat intertwined with general hiring and retention of individuals during the COVID-19 pandemic. It has been difficult to identify socio-demographically diverse candidates who are willing to potentially move to St. Louis in the midst of a global pandemic and are interested in staying on for a 2-year postdoc. Our diverse candidates who are willing to move here are often ones who are originally from the area. Having post-docs who are working 100% remotely up until very recently has made it difficult for them to feel like they are fully integrated into the Center and has limited the number of informal networking and mentorship experiences. We have learned that it takes much more time to intentionally seek out and hire socio-demographically diverse scholars and that it is important to broaden from the typical channels we use to disseminate job postings*.” |

**References**

1. Woodward EN, Matthieu MM, Uchendu US, Rogal S, Kirchner JE. The health equity implementation framework: proposal and preliminary study of hepatitis C virus treatment. Implementation Science. 2019;14(1):26.

2. Damschroder LJ, Aron DC, Keith RE, Kirsh SR, Alexander JA, Lowery JC. Fostering implementation of health services research findings into practice: a consolidated framework for advancing implementation science. Implementation science : IS. 2009;4:50.

3. Shelton RC, Chambers DA, Glasgow RE. An Extension of RE-AIM to Enhance Sustainability: Addressing Dynamic Context and Promoting Health Equity Over Time. Frontiers in public health. 2020;8(134).

4. Cohen D, McDaniel RR, Crabtree BF, Ruhe MC, Weyer SM, Tallia A, et al. A Practice Change Model for Quality Improvement in Primary Care Practice. Journal of Healthcare Management. 2004;49(3).

5. Feldstein AC, Glasgow RE. A practical, robust implementation and sustainability model (PRISM) for integrating research findings into practice. Joint Commission journal on quality and patient safety / Joint Commission Resources. 2008;34(4):228-43.

6. Glasgow RE, Vogt TM, Boles SM. Evaluating the public health impact of health promotion interventions: the RE-AIM framework. Am J Public Health. 1999;89(9):1322-7.

7. Aschbrenner KA, Kruse G, Emmons KM, Singh D, Barber-Dubois ME, Miller AM, et al. Stakeholder and Equity Data-Driven Implementation: a Mixed Methods Pilot Feasibility Study. Prevention science : the official journal of the Society for Prevention Research. 2022:1-11.

8. Gaias LM, Arnold KT, Liu FF, Pullmann MD, Duong MT, Lyon AR. Adapting strategies to promote implementation reach and equity (ASPIRE) in school mental health services. Psychology in the Schools. 2022;59(12):2471-85.
